# Letter from Dr. D. B. St. John Roosa

**Published:** 1897-04

**Authors:** D. B. St. John Roosa

**Affiliations:** 20 E. 30th St., New York


### Correspondence.

### LETTER FROM DR. D. B. ST. JOHN ROOSA.

*Graduated Tenotomy for the Relief of Asthenopia.— Use of Mydriatics in Determining Refractive Errors.— Comments on Dr. Rider's Paper.*

*To the Editor :*

Sir—I beg leave to express my interest in the article, appearing in the March number of your esteemed Journal, entitled, The Rational Treatment of Asthenopia, by Dr. Wheelock Rider, of Rochester. My interest is not entirely due to the well-narrated cases of asthenopia there reported, which have been treated by graduated tenotomy, but largely to the conclusion, in which Dr. Rider states that he wishes to place himself "on record in 1896 as saying that graduated tenotomy for the relief of asthenopia is, as a rule, irrational and dangerous."

As early as 1890, in a paper read before the Academy of Medicine,[1] and in an article in the *Ophthalmic Review*,[2] I formally called the attention of the profession to the erroneous doctrines so vociferously and widely proclaimed as to the cure of so-called muscular asthenopia by prisms and tenotomies. I urged, from long

1. March 20, 1890, in *Medical Record*, April 19, 1890.
2. *Ophthalmic Review*, October, 1890.

experience, the unreasonableness of hoping for anything from such practices. I also spoke on this subject in the ophthalmological section of the Berlin Congress of 1890, in English and German, without successful or scarcely any contradiction. Taval, at this meeting, confirmed my views from his earlier and better investigations.

I continued these demonstrations of the worthlessness of the treatment of asthenopia by prisms and tenotomies, in various articles published in various places, especially in the *Transactions of the Medical Society of the State of New York*, 1892, in the *Medical Record*,[3] and in the *New York Medical Journal.* I have also, in my work on the Eye (September, 1894,) given very fully my reasons for a belief identical with, or even more radical than that of Dr. Rider, of this irrational treatment, and which he has so well presented in his interesting paper. My voice on this subject has very often appeared to me to be that of " one crying in the wilderness,"—to have received very little attention, compared to what it deserved. It is only in our country that this "irrational" treatment of asthenopia has ever acquired any prevalence or prominence. It is now fast falling out of the consideration of ophthalmologists of experience, especially those who deal with large numbers of patients in eye hospitals, although few print their recantations. It is very unfortunate, in my judgment, that it still is in sufficient vogue to receive serious thought at the hands of men whose observations have been so extensive as those of the writer of the article I am commenting upon.

I cannot conclude my note of approbation of Dr. Rider's paper without expressing the hope that he will very soon agree with my teaching, also some years old, that a mydriatic is not necessarily used for the purpose of determining the refraction in all eyes, not presbyopic, as stated by him. I have repeatedly endeavored to show the use of a mydriatic to be scarcely ever necessary, even for young people, in the determination of the existence and degree of errors of refraction, and for ascertaining the origin of asthenopia. If the cornea be accurately measured,—as it now can be quickly and readily by every ophthalmologist,—it is very easy, by means of test glasses and the ophthalmoscope, to ascertain the degree of axial hypermetropia or myopia, and order the proper glass without the use of a mydriatic of any kind. If the patient is able to hold his or her eyes still enough to allow us to measure the cornea, the

3. November 26, 1892.

use of atropia is wholly unnecessary, except for the purpose of therapeutics, and these cases,—if we exclude inflammatory conditions—occur very infrequently.

I trust Dr. Rider will take another step forward, and emancipate himself from what I deem to be a troublesome, time-consuming, and not always safe method of determining the refractive power of the eye. D. B. ST. JOHN ROOSA.

20 E. 30th St., New York, March 19, 1897.

